# Prevalence, Antibiotic Susceptibility Profile, and Associated Risk Factors of *Salmonella* Isolate among Diarrheal Patients Visiting Dessie Referral Hospital, Northeast Ethiopia

**DOI:** 10.1155/2020/8834107

**Published:** 2020-10-22

**Authors:** Samuel Chane Teferi

**Affiliations:** College of Natural and Computational Sciences, Department of Biology, Salale University, Fitche, Ethiopia

## Abstract

Salmonellosis remains an important public health problem worldwide, particularly in the developing countries such as Ethiopia. A cross-sectional study was conducted to determine the prevalence, antibiotic susceptibility profile, and associated risk factors of *Salmonella* isolate among diarrheal patients who were visiting Dessie Referral Hospital, Dessie, Northeast Ethiopia, from November 2016 to January 2017. 384 stool samples were collected using sterile stool cups. Out of these, 20 (5.21%) were found to be positive for *Salmonella* species. The distribution of positive samples among the three age groups indicated that *Salmonella* species were predominantly prevalent in the age group of three months (0.25 years) to 4 years old patients. Abdominal pain, vomiting, watery consistency of stool, and 1–5 days of diarrhea were the clinical features that were significantly associated with salmonellosis. Eating raw vegetables and fruits, consumption of street-vended foods, cohabitation of animals, using water from the unprotected source, absence of latrine, and consumption of raw products of animals such as eggs and raw milk were the risk factors that were significantly associated with the prevalence of *Salmonella* species. The antibiotic sensitivity test was performed for the isolated *Salmonella* species against 5 currently recommended antibiotics. The antimicrobial sensitivity study carried out using the Kirby–Bauer disk diffusion method showed that 100% of *Salmonella* isolates were sensitive to ciprofloxacin and 80% sensitive to nalidixic acid, respectively. Among them, twenty isolated *Salmonella* species resistant to ampicillin, tetracycline, and trimethoprim-sulfamethoxazole were 100%, 85%, and 80%, respectively. This study revealed that *Salmonella* species were prevalent among diarrheal patients who were visiting Dessie Referral Hospital, and therefore, routine diagnosis of patients with diarrhea cases is required, and drugs must be prescribed after performing the sensitivity test, or checking the updated information on the local antibiotics pattern is always necessary.

## 1. Introduction

Diarrhea is a condition of having three or more loose or liquid bowel movement per day [1]. Human diarrheal diseases have been recognized since the beginning of civilization and remain one of the most prevalent public health problems of today. Diarrheal infection spreads through the ingestion of contaminated food or drinking water or person to person contact as a result of poor hygiene. According to [1], diarrheal disease is the second leading cause of death in children under five years old. Each year diarrhea kills around 525,000 under-five children. 12% from the estimated 3.6 million child deaths in 2010 were attributed to diarrheal diseases. The incidence of childhood diarrhea in Africa has also decreased from 4.2 to 3.3 episodes per child-year from 1990 to 2010, but sub-Saharan Africa still accounts for one-third of diarrheal episodes yearly (500 million of 1.7 billion worldwide), with the highest incidence among children of 6–11 months of age [2]. In Ethiopia, as in other sub-Saharan African countries, morbidity from diarrheal diseases is a serious health problem, and according to [3], *Salmonella* was one of the major causes of diarrhea in humans. Diarrhea in children is the second leading top ten causes of death that accounted 8% in Ethiopia [4].

Diarrhea can be caused by a wide variety of bacterial, viral, and protozoan pathogens. The most important of these are *Salmonella spp, Shigella spp, Vibrio cholera, Entamoeba histolytica, Norovirus, Giardia lamblia, Escherichia coli, Campylobacter jejuni, Cryptosporidium sp.,* and *Rotavirus* [5].

Salmonellosis, the disease caused by *Salmonella*, is one of the most frequently occurring foodborne diseases worldwide [6]. As a result, it continues to be a major health burden worldwide. In Ethiopia, 10.5% *Salmonella* prevalence was reported in Butajira [7], 6.2% *Salmonella* prevalence was reported in Jimma Health Center [8], a prevalence of 14.6% *Salmonella* infection was reported in Harar among patients who were admitted to hospital [9], 3.95% *Salmonella* infection was reported in children of Addis Ababa [10], and 2.5% was reported in Hawassa [11].

The majority of *Salmonella* cases occur as the result of ingesting contaminated food or water, but it can also be transmitted from person to person via the oral-fecal route. *Salmonella* can also be acquired by contact with domestic animals and their food products, farm animals or animals in petting zoo, and exotic pets such as turtles, hedgehogs, and iguanas [12].

A study in Ethiopia indicated that there is an increase in antimicrobial resistant in pathogenic bacteria causing diarrheal disease because of easy affordability of antibiotics in pharmacies and misuse [3, 13]. Therefore, antimicrobial resistance in bacterial pathogens causing diarrheal diseases makes it difficult to treat patients. In Dessie town, particularly the rural area of Dessie, there are a number of risk factors, which lead to gastrointestinal infections. For example, poor sanitary conditions, cattle living together with people, drinking raw milk and eating raw meat, and consumption of street-vended foods are common practices. These factors are directly or indirectly related to consumption of water and foods. According to [12], contaminated water and foods are responsible for initiating salmonellosis. So, the objective of this study was to investigate the prevalence, antibiotic susceptibility profile, and associated risk factors of *Salmonella* among diarrheal patients visiting Dessie Referral Hospital, Dessie town, Northeast Ethiopia.

## 2. Materials and Methods

### 2.1. Study Design

A cross-sectional study was carried out from November 2016 to January 2017 on diarrheal patients visiting Dessie Referral Hospital, Dessie. A pretested structured questionnaire was administered to patients or to their parents or guardians to collect information on sociodemographic and risk factors including sex, age, educational status, place of residence, source of drinking water, latrine usage, personal hygiene practice, and clinical features of the study subjects.

### 2.2. Study Population

The study included all diarrheal patients visiting the outpatient department of Dessie Referral Hospital. The sample size (*n*) was determined through a single proportion formula by taking an estimated prevalence of 0.5, since there is no previous investigation on the same title in the study area and margin of error 0.05. Sample size was determined using the following formula [14].

(1) $$\begin{array}{c} n=\frac{{\left(Z\alpha |/|2\right)}^{2}P\left(1-P\right)}{d^{2}}, \end{array}$$

where *n* is the number of sample size. Z*α*/2 is the standard score corresponding to 95% confidence level, i.e., 1.96. *P* is the prevalence of *Salmonella*. *D* is the marginal of error between the sample and the population. So, the calculated sample size for this study was 384.

### 2.3. Sampling Method

The sources of population were all diarrheal patients attending Dessie Referral Hospital. Those patients who had diarrhea and included in the sample were coded and assisted supervised by physicians or nurses in the outpatient department to fill the structured questionnaires. Then, fresh stool specimens were collected from diarrheal cases at Dessie Referral Hospital and transported using sterile stool cups to Dessie Regional Laboratory for analysis.

### 2.4. Isolation and Identification of *Salmonella* Species

About 1 g of stool sample was added into a tube containing selenite *F* broth. Selenite broth was incubated aerobically at 37°C for 18 hours [15]. For ideal isolation of *Salmonella*, two different selective media, general purpose medium with low sensitivity MacConkey agar and a high selective xylose-lysine desoxycholate agar (Oxoid, England), were used. A loopful of faecal suspension from enrichment media or selenite broth was subcultured on xylose-lysine desoxycholate agar and MacConkey agar. Then, plates were incubated aerobically at 35–37°C for 18–24 hours. Typical colorless colonies on MacConkey agar and colonies with a black center and a lightly transparent zone of reddish color due to the color change of the indicator were picked, and a series of biochemical tests were performed for further identification [15]. Those are the urease test (HiMedia, India), triple sugar iron agar (TSI) test (HiMedia, India), lysine iron agar (LIA) test (Oxoid, England), motility test (Oxoid, England), indole test (Oxoid, England), and citrate utilization test (Oxoid, England).

### 2.5. Antimicrobial Susceptibility Test

The antibiotic susceptibility profile of *Salmonella* isolates was determined for the commonly used antibiotics (Oxoid Ltd., Basingstoke, England). Each isolate was tested for the selected antimicrobial agents with their respective concentrations such as ampicillin (10 *μ*g), tetracycline (30 *μ*g), trimethoprim-sulfamethoxazole (1.25/23.75 *μ*g), nalidixic acid (30 *μ*g), and ciprofloxacin (100 *μ*g). Using a sterile wire loop, 3–5 well isolated colonies were picked and emulsified in 5 ml nutrient broth. The prepared turbidity was matched with a turbidity standard (0.5 McFarland) to have an equivalent suspension. A sterile swab was used to inoculate the suspension by streaking on the prepared and dried Mueller–Hinton agar plate evenly. It was then allowed to stay for 3–5 minutes. Sterile forceps were used to place the antimicrobial discs on the inoculated plates [16].

Within 30 minutes after applying the disc, the plates were incubated at 35°C for 16–18 hours. After incubation, diameters of the zone of inhibition was measured to the nearest millimeter using a metallic caliper, and the isolates were classified as sensitive, intermediate, and resistant according to the standardized table provided by [16]. The standard reference strain, *Escherichia coli* (American type culture collection 25922), was used as a control for the study.

### 2.6. Data Analysis

The data were entered and analyzed using the Statistical Package for Social Sciences (SPSS) version 16 software. The prevalence of *Salmonella* species was calculated as percentage, and results are presented in tables. Statistical significance of the association was measured by using the chi-square test (association between selected risk factors and *Salmonella* infection). *P* values less than 0.05 were considered as statistically significant.

### 2.7. Ethical Considerations

Institutional consent was acquired through communications made with Dessie Referral Hospital before conducting the study. The medical director of Dessie Referral Hospital was briefed first about the study before meeting with patients. Both oral and written informed consents were obtained from the parents or guardians of the study subjects before administration of the study. The participation of patients in this study was purely a voluntary activity, and their right not to participate was respected. Issues of confidentiality and anonymity were also maintained.

## 3. Results and Discussion

Three hundred eighty-four (*n* = 384) diarrheal patients attending the outpatient department of Dessie Referral Hospital were examined for *Salmonella* spp during the three-month study period. Distribution of the study participants by sex and age is shown in Table 1. Age group category was based on the Dessie Regional Health Bureau data record system on diarrheal diseases prevalence, where 0–4, 4–14, and ≥15 age groups are reported as under-five children, young children, and adults, respectively.

Highest frequency of diarrheal diseases occurred in the age group of 3 months to four years (Table 1). This was consistent with [2] who reported a highest incidence among children of 6–11 months of age.

### 3.1. Prevalence of *Salmonella* Species

The prevalence of *Salmonella* isolates in stool samples of this study, 5.21% (20/384), is comparable to the findings of previous studies made in China by [17] who reported a prevalence of 5.8% and 4.8% *Salmonella* in the years 2006 and 2007, respectively, and 5.3% a study performed at Jimma and Addis Ababa, Ethiopia [13].

On the other hand, a lower prevalence was reported by [18] from Djibouti who reported a prevalence rate of 2.9% and 3.3% from Lagos, Nigeria [19], 3% Botswana [20], and 1.08% [21], 2.5% [11], and 3.95% [10] from Ethiopia. However, the isolation rate in this study was found to be lower than the 6.2% *Salmonella* prevalence reported by [8], a study conducted on the prevalence of intestinal parasite, *Shigella* and *Salmonella* species among diarrheal children in Jimma health center, Jimma, southwest Ethiopia, and much lower than the 10.5% and 14.6%, 16.7%, and 17% prevalence of *Salmonella* reported by [7, 9, 22, 23] in Ethiopia, Mexico, and Nigeria, respectively.

The prevalence of *Salmonella* infection by age and sex in the present study is also shown in Table 1. As can be seen from the table, *Salmonella* infection was seen in both male and female patients, but it was statically insignificant with 0.800 of the chi-square value and *P* value of 0.371. This implies that both sexes are equally at risk of *Salmonella* infection. Among the 20 patients infected with *Salmonella* species, 12 (60%) were females and 8 (40%) were males, which gave an overall male to female ratio of 1:1.5.

As indicated in Table 1, *Salmonella* were isolated in all age groups, and a statistically significant difference existed between the prevalence of the different age groups (*P*=0.022). Out of 20 positive *Salmonella* isolates, more *Salmonella* infection (12/20 or 60%) was observed in children of 0–4 years of age than in those young children aged between 5 and 14 years (30%) and adults above 15 years (10%).

### 3.2. Association of *Salmonella* Infection and Identified Risk Factors

The association of *Salmonella* infection and prevalence of *Salmonella* spp. are shown in Table 2. The data generally show that infection with *Salmonella* spp. is associated significantly with the absence of latrine (*X*^2^ = 7.200, *P*=0.007), drinking unprotected water (*X*^2^ = 5.000, *P*=0.025), consumption of egg (*X*^2^ = 5.000, *P*=0.025), cohabitation with domestic animals (*X*^2^ = 7.200, *P*=0.007), consumption of raw milk (*X*^2^ = 9.800, *P*=0.002), consumption of raw vegetables and fruits (*X*^2^ = 16.200, *P*=0.000), and consumption of street-vended foods (*X*^2^ = 12.800, *P*=0.000).

In this study, there was a significant link between the absence of latrine and infection with *Salmonella*. Contracting salmonellosis and poor living and housing conditions was significantly associated. This finding was typical to the situation, throughout the developing world [24].

Association of drinking from the unprotected water source and prevalence of salmonellosis (*X*^2^ = 5.000 and *P*=0.025) was significant. From 20/384 positive cases for salmonellosis, those who used protected water had less risk of contracting salmonellosis than those who used from the unprotected source (Table 2). This can be due to the high level of contamination of water by *Salmonella* from the environment. The finding was in line with [25] who reported that the transmission of *Salmonella typhi* in humans was associated with the ingestion of contaminated water. Moreover, a study has shown that exposure to contaminated water is known to be associated with diarrhea caused by ingestion of microorganisms [26].

*Salmonella* spp. infection was shown to be significantly associated with those who had domestic animals (cohabitation); the present finding was in line with [9] from Harar, Ethiopia. This finding suggests the fact that *Salmonella* spp. are important human and animal pathogens and acquired from contact with domestic animal and their food products [12]. Besides, the aquatic environment is liable to contamination through colistin-treated animal faeces via the use of manures in feeding farmed fish [27].

Raw milk and raw milk products consumption from a dairy may result in infection by *Salmonella* and many other pathogenic bacteria [28]. The present finding also revealed a significant association between raw milk consumption and contracting of *Salmonella* infection. This is comparable to the findings in Pennsylvania who reported the transmission of enteric pathogen infections to humans via the consumption of raw milk [29].

In the present study, an association between salmonellosis and consumption of raw vegetables and fruits from unhygienic sources were significant (Table 2). In recent years, fresh produce such as fruits and vegetables have gained concern as vehicles of transmission where contamination can occur at multiple steps along the food chain [30].

The prevalence of *Salmonella* spp. was also associated with the consumption of street-vended foods, which was consistent with a report from Ghana by [9, 31] from Harar, Ethiopia. This might be due to street-vended foods sold under unhygienic conditions in the study area. *Salmonella* has been known as the most important foodborne pathogen, which can infect humans via consuming contaminated food [32]. Moreover, identifying genetic subpopulations and understanding their epidemiology may contribute to efforts invested in the prevention and mitigation of these important foodborne pathogens [33].

According to [34], consumption of contaminated products such as meat and eggs initiate salmonellosis in human. In the present study, a significant association (*X*^2^ = 5.000, *P*=0.025) was found between consumption of raw eggs and *Salmonella* infection. Those patients who consumed raw egg were likely contracting salmonellosis than those who did not consume such products. Moreover, according to [35], human and poultry isolates bore more antimicrobial resistance and virulence genes, and the meat pathway may be an important source of human infection by some clades of *Salmonella enteritidis* ST11 in East Africa, and according to [36], the emergence and spread of new strains of zoonotic bacteria, such as multidrug resistant (MDR) *Salmonella infantis*, represent a growing health risk for humans in and outside Europe due to foodborne infections of poultry meat origin.

There was no statistically significant association between residence, educational status, presence of domestic animal, and raw meat on one hand and infection with *Salmonella* on the other hand as indicated in Table 2.

### 3.3. Association between the Prevalence of *Salmonella* spp. and Clinical Features

Association between the prevalence of *Salmonella* spp. and clinical features are summarized in Table 3. In this study, out of twenty *Salmonella*-positive patients, the majority had abdominal pain followed by vomiting and fever, which accounted for 90%, 80%, and 70% of the patients, respectively. This result was comparable with the study conducted in Harar by [9] who reported 85.7% abdominal pain and [11] showed 50% abdominal pain. There was a significant association between abdominal pain, vomiting, and *Salmonella* infection (*X*^2^ = 12.800, 7.200 and *P*=0.000,  0.007, respectively), whereas there was no significant association in fever (Table 3).

The present study showed that there was a significant association between consistency of stools, duration of diarrhea, and positivity of *Salmonella* infection (*X*^2^ = 13.600, 7.600 and *P*=0.004,  0.022, respectively) as the majority 60% (12) of the stool samples were watery diarrhea in contrast to [37] in Harar who reported that no *Salmonella* was isolated from watery diarrhea. But the present study is comparable with [9], a study performed in Harar who reported 28.6% watery diarrhea, and 82.4% *Salmonella* was isolated from watery diarrhea in a study performed in Addis Ababa [38].

The remaining 20% (4), 10% (2), and 10% (2) of the isolated stool samples were mucoid, bloody, and mixed (blood and mucus), respectively (Table 3). This may reflect the underlying variation in signs and symptoms with strain differences from place to place.

Out of the twenty isolates, 12 (60%) were having 1–5 days duration of diarrhea, which is consistent with [11] who reported 75% and [9] who reported 71.4% of the isolate was from 1 to 5 days. Whereas the remaining 30% and 10% of *Salmonella* isolate were 6–10 and 11–15 days of diarrhea, respectively. The use of clinical signs and symptoms is therefore very important in helping to identify patients with salmonellosis.

### 3.4. Antimicrobial Susceptibility Test

The antimicrobial susceptibility tests were performed on all *Salmonella* isolates using the disk diffusion method, and the results are presented in Table 4. Among 20 *Salmonella* isolates, 20 (100%), 17 (85%), 16 (80%), and 3 (15%) were resistance to ampicillin, tetracycline, trimethoprim-sulphamethoxazole, and nalidixic acid, respectively. But all *Salmonella* isolates showed 100% susceptibility to ciprofloxacin.

3 (15%), 2 (10%), and 1 (5%) of the isolate showed an intermediate resistance to tetracycline, trimethoprim-sulfamethoxazole, and nalidixic acid, respectively. Even though most of the isolate showed different resistance patterns, 16 (80%) and 2 (10%) tested isolates were susceptible to nalidixic acid and trimethoprim-sulfamethoxazole, respectively. The antimicrobial resistance in *Salmonella* is one of the main concerns of its infection in humans [32].

As can be seen from Table 4, *Salmonella* were resistance to ampicillin (100%), tetracycline (85%), and trimethoprim-sulfamethoxazole (80%), which was comparable to the findings in Harar (100%, 100%, and 85.7%, respectively) as reported by [9] and 100% and 86.8% resistant to ampicillin and trimethoprim-sulfamethoxazole as reported by [39] in Pakistan. Moreover, a study from Addis Ababa reported that 81.2%, 94.5%, and 75.7% of the isolate was resistant to ampicillin, tetracycline, and trimethoprim-sulfamethoxazole, respectively [38]. In contrast to the present study, all were found to be susceptible to ampicillin, tetracycline, and trimethoprim-sulfamethoxazole in a study at Hawassa by [11].

The present finding was higher than the previous studies reported by [7], which showed that a high frequency of resistance in *Salmonella* isolates was observed to tetracycline (52.5%), cotrimoxazole (37.5%), and ampicillin (60%). Moreover, 80% of the isolate had resistance to ampicillin according to [10]. The rate of resistance of *Salmonella* isolates to tetracycline in present study (85%) was higher than in Harar (71.4%) [37] and in Mozambique (15%) [40].

Resistance to ampicillin and trimethoprim-sulfamethoxazole in the present study was also higher than those reported from Brazil, where only 88.8% and 56.8% were found to be resistant, respectively [39]. But 86.4% resistant to tetracycline is comparable with the present study.

The increased resistance towards ampicillin (100%), tetracycline (85%), and trimethoprim-sulfamethoxazole (80%) in this study might be due to misuse of those antibiotics in the study area. It was revealed that antibiotic resistance of *Salmonella* isolates from diarrheic children in Southeastern Africa was conferred by TEM-like *β*-lactamases for ampicillin, *floR* genes, and CAT activity for chloramphenicol, *tetA* genes for tetracycline, and *dfrA*1 genes for trimethoprim-sulfamethoxazole/cotrimoxazole [40].

In the present study, 100% and 80% of *Salmonella* isolates were shown to be susceptible to ciprofloxacin and nalidixic acid, respectively, and they can be used to treat salmonellosis in the study area. The present finding was in line with a study conducted in Harar by [9].

Table 5 shows findings from different regions and periods, such as central Ethiopia (Addis Ababa), Southern Ethiopia (Butajira), southwest Ethiopia (Jimma), Eastern Ethiopia (Harar), and Dessie (the present study). The data revealed that the highest percentages of ampicillin and tetracycline resistant *Salmonella* isolates were reported from Harar (100% each), Addis Ababa (81.3% and 94.5%) in 2008, and Dessie (100% and 85%). Similarly, an increased resistance of trimethoprim-sulphamethoxazole was documented in Jimma and Addis Ababa [13], Dessie (the present study), and Addis Ababa [38], where 80.5%, 80%, and 75.7% have been shown to be resistant to this antibiotic, respectively. Resistance against trimethoprim-sulfamethoxazole and tetracycline was reported from Jimma [3] and Jimma and Addis Ababa [13] where 40.7% and 39.8%, respectively, were found to be resistant to those antibiotics.

## 4. Conclusion

The result of the present study showed that the prevalence of salmonellosis among diarrheal patients who visited Dessie Referral Hospital, Dessie, Northeast Ethiopia, from November to January 2017 was 5.21%. This prevalence rate of *Salmonella* species is associated with drinking unprotected water, consumption of raw vegetable and fruits, consumption of raw meat, and absence of latrine in the households. Relatively, its prevalence was lower for those study subjects that had safe and adequate drinking water and proper excreta disposal systems.

Prevention of salmonellosis depends primarily on measures that prevent the spread of the organism in the community and prevent the spread from person to person through washing hand with soap after toilet, by drinking protected water, and proper handling of foods. These measures are not only used for salmonellosis but are also used for other diarrheal diseases. In nutshell, creating awareness about transmission and risk factors of *Salmonella* is necessary.

In this study, 100% of the isolate was susceptible to ciprofloxacin, so it may be used for the treatment of salmonellosis. Besides, tetracycline, ampicillin, and cotrimoxazole should not be used for the treatment of *Salmonella* infection unless culture and sensitivity tests are performed prior to treatment.

## 5. Recommendations

The following recommendations are made based on the finding:

  The present study did not differentiate *Salmonella* isolate into the species level. Therefore, further studies that aim to differentiate serogroups and serotypes of *Salmonella* are needed.
  The present study was conducted in a short period of time from November to January 2017. Additional study is needed to assess the seasonal variation in prevalence and antimicrobial resistance patterns of *Salmonella* species.
  Raw eggs, raw vegetables and fruit, and milk should not be consumed in order to limit the spread of salmonellosis
  Pretreatment of water obtained from unprotected sources prior to consumption, the need to improve the hygienic conditions of street vended foods, and reduce and remove too many contact (living with animals) in order to limit the spread of salmonellosis

## Figures and Tables

**Table 1:** Prevalence of *Salmonella* spp. and sociodemographic characteristics of the study participants (*n* = 384).

Sociodemographic characteristics		Total numbers examined	No. of positives (%)	*P* values
Age (years)	0.25–4	180	12 (60)	0.022^*∗∗∗*^
	5–14	120	6 (30)	
	>15	84	2 (10)	
Gender	Male	181	8 (40)	0.371
	Female	203	12 (60)	
Place of residence	Urban	171	7	0.180
	Rural	213	13	
Educational status	Literate	142	12	0.371
	Illiterate	242	8	

**Table 2:** Prevalence of *Salmonella* spp. and associated risk factors in the study area in 2016/17.

Factors	*Salmonella* species		*X* ^2^	Df	*P* value
	Positive (%)	Negative (%)			
Residence					
Urban	7 (4.1)	164 (95.9)	1.800	1	0.180
Rural	13 (6.1)	200 (93.9)			
Educational status					
Literate	12 (8.5)	130 (91.5)	0.800	1	0.371
Illiterate	8 (3.3)	234 (96.7)			
Latrine					
Present	3 (1.4)	210 (98.6)	7.200	1	0.007^*∗∗∗*^
Absent	17 (10)	154 (90)			
Drinking water					
Protected	5 (2.5)	194 (97.5)	5.000	1	0.025^*∗∗∗*^
Unprotected	15 (8.1)	170 (91.9)			
Domestic animals					
Present	14 (6.1)	214 (93.9)	3.200	1	0.074
Absent	6 (3.8)	150 (96.2)			
Domestic animal house					
Separate	4 (2.2)	174 (97.8)	7.200	1	0.007^*∗∗∗*^
Cohabit	16 (7.8)	190 (92.2)			
Raw milk					
Used	17 (14.5)	100 (85.5)	9.800	1	0.002^*∗∗∗*^
Unused	3 (1.1)	264 (98.9)			
Raw meat					
Used	13 (6.1)	200 (93.9)	1.800	1	0.180
Unused	7 (4.1)	164 (95.9)			
Raw vegetables and fruits					
Used	19 (5.9)	302 (94.1)	16.200	1	0.000^*∗∗∗*^
Unused	1 (1.6)	62 (98.4)			
Consumption of street-vended foods					
Used	18 (8.5)	193 (91.5)	12.800	1	0.000^*∗∗∗*^
Unused	2 (1.2)	171 (98.8)			
Raw egg					
Used	15 (6)	234 (94)	5.000	1	0.025^*∗∗∗*^
Unused	5 (3.7)	130 (96.3)			

^*∗∗∗*^Significant at *P* < 0.05; Df, degree of freedom; *X*^2^, chi-square.

**Table 3:** Associations of *Salmonella* infection and clinical features in diarrheal patients.

Symptoms	Positive (%)	Negative (%)	*X* ^2^	Df	*P* values
Fever					
Yes	14 (5.5)	238 (94.5)	3.200	1	0.074
No	6 (4.5)	126 (95.5)			
Vomiting					
Yes	16 (6.8)	220 (93.2)	7.200	1	0.007^*∗∗∗*^
No	4 (2.7)	144 (97.3)			
Abdominal pain					
Yes	18 (6.9)	241 (93.1)	12.800	1	0.000^*∗∗∗*^
No	2 (1.6)	123 (98.4)			
Consistency of stool					
Watery	12 (6.9)	162 (93.1)	13.600	3	0.004^*∗∗∗*^
Bloody	2 (4.8)	40 (95.2)			
Mucoid	4 (4)	96 (96)			
Mixed (blood and mucus)	2 (2.9)	66 (97.1)			
Duration of diarrhea (days)					
1–5	12 (3.9)	294 (96.1)	7.600	2	0.022^*∗∗∗*^
6–10	6 (9.7)	56 (90.3)			
11–15	2 (16.7)	10 (83.3)			
≥16 days	0.0	4 (100)			

^*∗∗∗*^Significant at *P* < 0.05; *X*^2^, chi-square; Df, degree of freedom.

**Table 4:** Drug resistance/susceptibility pattern of *Salmonella* species isolated in thepresent study (*n* = 20).

Susceptibility	AMP no. (%)	TRA no. (%)	SXT no. (%)	NA no. (%)	CI no. (%)
*R*	20 (100%)	17 (85%)	16 (80%)	3 (15%)	0
*I*	0	3 (15%)	2 (10%)	1 (5%)	0
*S*	0	0	2 (10%)	16 (80%)	20 (100%)

AM, ampicillin; CI, ciprofloxacin; SXT, trimethoprim-sulphamethoxazole; NA, nalidixic acid; TRA, tetracycline; *S,* sensitive; *I,* intermediate; *R,* resistance.

**Table 5:** Antimicrobial resistance patterns of *Salmonella* in the present study and similar other research studies in different parts of Ethiopia.

Study area with respective study periods							
Antibiotic	Jimma^1^	Addis Ababa^2^	Harar^3^	Jimma and Addis Ababa^4^	Harar^5^	Butajira^6^	Dessie^*∗*^
AM	59.3	81.3	100	82.3	100	60	100
CI	Nd	Nd	Nd	0.9	28	2.5	0.0
SXT	40.7	75.7	Nd	80.5	Nd	37.5	80
NA	8.5	37.8	Nd	8.0	3	5	15
TRA	59.3	94.5	71.4	39.8	100	52.5	85

1, [3]; 2, [38]; 3, [37]; 4, [13]; 5, [9]; 6, [7]; ^*∗*^the present study; Nd, no data; AM, ampicillin; CI, ciprofloxacin; SXT, trimethoprim-sulfamethoxazole; NA, nalidixic acid; TRA, tetracycline.

## Data Availability

The dataset used to support the findings of this study is available from the author upon request.
